# Clinical criteria for a limbic-predominant amnestic neurodegenerative syndrome

**DOI:** 10.1093/braincomms/fcae183

**Published:** 2024-07-17

**Authors:** Nick Corriveau-Lecavalier, Hugo Botha, Jonathan Graff-Radford, Aaron R Switzer, Scott A Przybelski, Heather J Wiste, Melissa E Murray, Robert Ross Reichard, Dennis W Dickson, Aivi T Nguyen, Vijay K Ramanan, Stuart J McCarter, Bradley F Boeve, Mary M Machulda, Julie A Fields, Nikki H Stricker, Peter T Nelson, Michel J Grothe, David S Knopman, Val J Lowe, Ronald C Petersen, Clifford R Jack, David T Jones

**Affiliations:** Department of Neurology, Mayo Clinic, Rochester, MN 55905, USA; Department of Psychiatry and Psychology, Mayo Clinic, Rochester, MN 55905, USA; Department of Neurology, Mayo Clinic, Rochester, MN 55905, USA; Department of Neurology, Mayo Clinic, Rochester, MN 55905, USA; Department of Neurology, Mayo Clinic, Rochester, MN 55905, USA; Department of Quantitative Health Sciences, Mayo Clinic, Rochester, MN 55905, USA; Department of Quantitative Health Sciences, Mayo Clinic, Rochester, MN 55905, USA; Department of Neuroscience, Mayo Clinic, Jacksonville, FL 3224, USA; Department of Laboratory Medicine and Pathology, Mayo Clinic, Rochester, MN 55905, USA; Department of Neuroscience, Mayo Clinic, Jacksonville, FL 3224, USA; Department of Laboratory Medicine and Pathology, Mayo Clinic, Rochester, MN 55905, USA; Department of Neurology, Mayo Clinic, Rochester, MN 55905, USA; Department of Neurology, Mayo Clinic, Rochester, MN 55905, USA; Department of Neurology, Mayo Clinic, Rochester, MN 55905, USA; Department of Psychiatry and Psychology, Mayo Clinic, Rochester, MN 55905, USA; Department of Psychiatry and Psychology, Mayo Clinic, Rochester, MN 55905, USA; Department of Psychiatry and Psychology, Mayo Clinic, Rochester, MN 55905, USA; Department of Pathology, University of Kentucky, Lexington, KY 40506, USA; CIEN Foundation/Queen Sofia Foundation Alzheimer Center, Madrid, Spain; Wallenberg Center for Molecular and Translational Medicine and Department of Psychiatry and Neurochemistry, University of Gothenburg, Gothenburg, Sweden; Department of Neurology, Mayo Clinic, Rochester, MN 55905, USA; Department of Radiology, Mayo Clinic, Rochester, MN 55905, USA; Department of Neurology, Mayo Clinic, Rochester, MN 55905, USA; Department of Radiology, Mayo Clinic, Rochester, MN 55905, USA; Department of Neurology, Mayo Clinic, Rochester, MN 55905, USA; Department of Radiology, Mayo Clinic, Rochester, MN 55905, USA

**Keywords:** amnestic syndrome, limbic age-related 43 encephalopathy, Alzheimer’s disease, behavioural neurology, limbic-predominant amnestic neurodegenerative syndrome

## Abstract

Predominant limbic degeneration has been associated with various underlying aetiologies and an older age, predominant impairment of episodic memory and slow clinical progression. However, the neurological syndrome associated with predominant limbic degeneration is not defined. This endeavour is critical to distinguish such a syndrome from those originating from neocortical degeneration, which may differ in underlying aetiology, disease course and therapeutic needs. We propose a set of clinical criteria for a limbic-predominant amnestic neurodegenerative syndrome that is highly associated with limbic-predominant age-related TDP-43 encephalopathy but also other pathologic entities. The criteria incorporate core, standard and advanced features, including older age at evaluation, mild clinical syndrome, disproportionate hippocampal atrophy, impaired semantic memory, limbic hypometabolism, absence of neocortical degeneration and low likelihood of neocortical tau, with degrees of certainty (highest, high, moderate and low). We operationalized this set of criteria using clinical, imaging and biomarker data to validate its associations with clinical and pathologic outcomes. We screened autopsied patients from Mayo Clinic and Alzheimer’s Disease Neuroimaging Initiative cohorts and applied the criteria to those with an antemortem predominant amnestic syndrome (Mayo, *n* = 165; Alzheimer’s Disease Neuroimaging Initiative, *n* = 53) and who had Alzheimer’s disease neuropathological change, limbic-predominant age-related TDP-43 encephalopathy or both pathologies at autopsy. These neuropathology-defined groups accounted for 35, 37 and 4% of cases in the Mayo cohort, respectively, and 30, 22 and 9% of cases in the Alzheimer’s Disease Neuroimaging Initiative cohort, respectively. The criteria effectively categorized these cases, with Alzheimer’s disease having the lowest likelihoods, limbic-predominant age-related TDP-43 encephalopathy patients having the highest likelihoods and patients with both pathologies having intermediate likelihoods. A logistic regression using the criteria features as predictors of TDP-43 achieved a balanced accuracy of 74.6% in the Mayo cohort, and out-of-sample predictions in an external cohort achieved a balanced accuracy of 73.3%. Patients with high likelihoods had a milder and slower clinical course and more severe temporo-limbic degeneration compared to those with low likelihoods. Stratifying patients with both Alzheimer’s disease neuropathological change and limbic-predominant age-related TDP-43 encephalopathy from the Mayo cohort according to their likelihoods revealed that those with higher likelihoods had more temporo-limbic degeneration and a slower rate of decline and those with lower likelihoods had more lateral temporo-parietal degeneration and a faster rate of decline. The implementation of criteria for a limbic-predominant amnestic neurodegenerative syndrome has implications to disambiguate the different aetiologies of progressive amnestic presentations in older age and guide diagnosis, prognosis, treatment and clinical trials.

## Teaser

Corriveau-Lecavalier *et al*. propose clinical criteria for a limbic-predominant amnestic neurodegenerative syndrome, which refers to a neurologic syndrome of a slow and predominant amnestic syndrome localizing to the degeneration of the limbic system. Limbic-predominant amnestic neurodegenerative syndrome is agnostic to molecular pathology, although highly associated with transactive response DNA-binding protein 43.

## Graphical Abstract

**Graphical Abstract:**
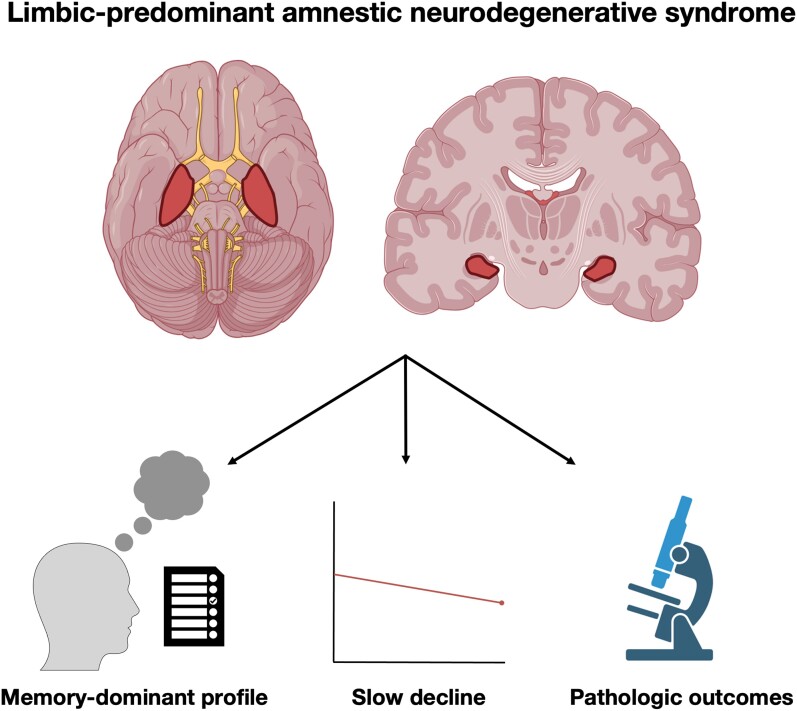

## Introduction

Selective and predominant degeneration of the limbic system has been associated with an older age, predominant and circumscribed episodic memory impairment and slow clinical course.^1-5^ Various underlying aetiologies are known to cause degeneration of the limbic system, the most frequent being limbic-predominant age-related TDP-43 encephalopathy neuropathological change (LATE-NC), which first involves the amygdala, followed by the hippocampus, and then the middle frontal gyrus,^1,6-9^ and is found in ∼40% of autopsied brains beyond age 85 years.^10,11^ Other rare neuropathologic findings have been associated with neurodegeneration of the limbic system, including argyrophilic grain disease, vascular disease and rare forms of limbic Alzheimer’s disease.^12-18^ By contrast, amnestic syndromes originating from neocortical degeneration are in vast majority caused by Alzheimer’s disease neuropathological changes (ADNCs) and often present with non-memory features, a more rapid clinical course and a relatively younger age than patients with predominant limbic degeneration.^2-4,19^ However, the recognition of different aetiologies driving predominant amnestic syndromes in clinical practice has been hampered by the significant overlap in clinical features associated with predominant limbic versus neocortical degeneration. This endeavour is further complicated by the fact that the most common cause of limbic degeneration, namely, LATE-NC, is frequently comorbid with other neuropathologies including amyloid plaques and tau tangles, i.e. ADNC, with which LATE-NC partially shares pathogenic mechanisms.^9,15,20-22^ This calls for much-needed clinical criteria to identify predominant amnestic syndromes caused by a predominant degeneration of the limbic system and to distinguish such syndromes from those with a predominant neocortical presentation, which may differ in terms of underlying aetiology, disease course and therapeutic needs. The current work aims to define and establish clinical criteria for the neurological syndrome associated with the selective degeneration of the limbic system, which we term limbic-predominant amnestic neurodegenerative syndrome (LANS). It is important to mention at the outset of this investigation that while LANS is associated with various underlying aetiologies, we primarily focus on LATE-NC in the context of this study to provide construct validity to the LANS criteria given the much higher frequency of LATE-NC in old age, its frequent comorbidity with ADNC^1,11,12,23^ and the far more extensive research that has been done on LATE-NC compared to other pathologies associated with limbic degeneration.

Several efforts based on retrospective clinicopathological studies have aimed to characterize the clinical profile of individuals who had predominant limbic degeneration and LATE-NC at autopsy and to disambiguate this profile from those who had ADNC without LATE-NC. The most common finding across studies is that episodic memory impairment dominates the clinical profile of individuals with LATE-NC, while cognitive functions functionally associated with neocortical areas (e.g. visuospatial processing) are relatively preserved. Studies that evaluated the longitudinal trajectory of cognitive impairment in LATE-NC consistently found a milder and slower decline of episodic memory and global function relative to ADNC and a steeper rate of decline in patients with evidence for both diseases, i.e. ADNC/LATE-NC. Evidence of semantic memory impairment in LATE-NC is measured through object naming, verbal fluency and knowledge of famous faces and events.^2,19,24^ For instance, a recent study from our group showed that an important feature of object naming failure in individuals with LATE-NC is loss of semantic memory, while word retrieval difficulties are more specific in patients with ADNC.^25^ However, these studies either did not assess semantic memory impairment in the context of a predominant amnestic syndrome, directly compared patients with LATE-NC versus ADNC or accounted for global severity of impairment, thus rendering the operationalization of semantic memory impairment in amnestic syndromes caused by limbic degeneration challenging. Overall, evidence supports a clinical profile characterized by a relatively isolated amnestic syndrome with an indolent progression in patients with LATE-NC relative to ADNC, in addition to semantic memory impairment, although less documented.

*In vivo* neuroimaging markers of autopsy-confirmed LATE-NC have been described. The hippocampus is a known locus of LATE-NC pathology. For example, MRI studies have repeatedly found smaller hippocampal volume and faster rates of hippocampal atrophy associated with LATE-NC when accounting for the extent of ADNC.^21,26-28^ Hippocampal sclerosis (severe cell loss and gliosis in the hippocampal formation, often with accentuated atrophy) is a frequent finding at autopsy associated with LATE-NC.^21,23,29^ In fact, studies have described patients with a dense amnestic syndrome who were either tau-negative on PET imaging or had TDP-43–related hippocampal sclerosis with minimal ADNC at autopsy.^30,31^ This strongly supports the hypothesis that LATE-NC is an independent driver of hippocampal atrophy that is sufficient to cause a progressive amnestic syndrome.

Fluorodeoxyglucose (FDG)-PET has also been proven useful for delineating patterns of involvement associated with LATE-NC and has the potential to objectively index impaired limbic function in the setting of preserved neocortical function. Botha *et al*.^28^ first developed the inferior-to-medial temporal (IMT) ratio as a marker of TDP-43–related hippocampal sclerosis in tau-negative amnestic dementia, where such patients exhibit prominent medial temporal lobe hypometabolism relative to the lateral temporal lobe, while the opposite pattern is typically associated with ADNC. Buciuc *et al*.^22^ subsequently showed that this marker is specific and sensitive to advanced LATE-NC regardless of hippocampal sclerosis status. A recent study by Grothe *et al*.^31^ showed that autopsy-confirmed cases of LATE-NC exhibit prominent temporo-limbic hypometabolism compared to those with ADNC and that independent patients with a clinical diagnosis of Alzheimer’s disease dementia showing this ‘LATE-NC–like’ pattern had an older age at evaluation, a memory-dominant cognitive impairment profile, a slower clinical course and lower Alzheimer’s disease biomarkers levels. These findings position temporo-limbic–predominant hypometabolism, in addition to disproportionate hippocampal atrophy on MRI, as a promising candidate for the *in vivo* identification of individuals with predominant limbic degeneration highly associated with underlying LATE-NC and for distinguishing such patients from those with neocortical degeneration and ADNC as the primary driver of their symptoms.

Despite the significant advances in identifying *in vivo* features indicative of limbic degeneration, especially in the context of LATE-NC, clinically applicable criteria of a predominant amnestic syndrome driven by a pattern of limbic-predominant degeneration do not currently exist. The *in vivo* identification of patients with a high likelihood of limbic degeneration as the primary driver of their symptoms has relevance for symptom management and prognosis and prediction of underlying aetiology, which are thought to differ from other amnestic syndromes associated with predominant neocortical degeneration. The detection of causes for predominant amnestic symptoms is also highly relevant in this era of emerging disease-modifying therapies to prevent patients from being inadvertently treated with inappropriate therapies,^32,33^ given that several non–Alzheimer’s disease aetiologies are associated with limbic degeneration.

We propose a set of clinical criteria for LANS. LANS is defined as a degenerative neurologic syndrome in that it refers to a set of clinical signs and symptoms associated with a requisite functional neuroanatomic localization, i.e. the limbic system. While the definition of LANS is agnostic to molecular pathology, this syndrome is highly associated with LATE-NC but also other less common neuropathologic entities as mentioned earlier. In the absence of one-to-one mapping with a single underlying pathology and lack of clinically applicable *in vivo* biomarker of TDP-43, LANS can be framed as a clinico-radiological, or neurologic, entity rather than clinicopathologic.^34,35^ While the current investigation focuses on LANS associations with LATE-NC, data on other neuropathologies are provided in Supplementary material. The LANS criteria include core, standard and advanced criteria that can be measured *in vivo* along with levels of certainty that clinical symptoms are caused by the predominant degeneration of the limbic system (described in Box 1). We operationalized this set of criteria based on clinical, imaging and biomarker features that can be obtained in current clinical practice for evaluating neurologic symptoms. It is important to specify that the LANS criteria and associated likelihoods are meant to guide decision-making in clinical practice. The quantitative operationalization of the LANS criteria described in the context of this specific study is meant to provide construct validity to the criteria by retrospectively applying it to a cohort of autopsied patients from the Mayo Clinic and Alzheimer’s Disease Neuroimaging Initiative (ADNI) cohorts. This operationalization thus serves a validation purpose in the context of current and future studies and is not meant to be concretely applied in clinical practice. For example, we recommend that current clinical standards for interpreting brain imaging be used rather than quantitative metrics that are not routinely available (see Box 1).

Box 1 **LANS**

Clinical criteria

‘Core clinical features’

Must present with a slow, amnestic, predominant neurodegenerative syndrome (insidious onset with gradual progression over 2 or more years) without another condition that better accounts for the clinical deficits.

‘Standard supportive features’

- Older age at evaluation (generally ≥75 years old)
- Mild clinical syndrome with largely preserved neocortical-predominant functions
- Hippocampal atrophy out of proportion to syndrome severity
- Impaired semantic memory in the setting of a mild syndrome

‘Advanced supportive features’

- Limbic hypometabolism and absence of neocortical degenerative pattern on FDG-PET imaging
- Low likelihood of significant neocortical tau pathology

‘Degree of certainty’

- Low likelihood: meets core features and ≤2 standard features
- Moderate likelihood: meets core features and ≥3 standard features or meets core features and ≥2 standard and 1 advanced features
- High likelihood: meets core features, ≥3 standard features and 1 advanced feature or meets core features, ≥2 standard features and 2 advanced features
- Highest likelihood: meets all core, standard and advanced features

## Materials and methods

### Participants

Two clinicopathological cohorts were used for this study. The primary cohort came from the Mayo Clinic Study of Aging and Alzheimer’s Disease Research Center research programmes from Mayo Clinic, Rochester (now referred to as ‘Mayo cohort’). The second cohort came from the ADNI cohort. We screened all autopsied patients from these two cohorts (Mayo cohort, *n* = 922; ADNI cohort, *n* = 93) and included those with an antemortem history of a predominant and progressive amnestic syndrome. This was defined by a clinical diagnosis of amnestic Alzheimer’s-type dementia or single- or multi-domain amnestic mild cognitive impairment at baseline according to widely accepted criteria.^36-38^ Specifically, an amnestic syndrome was defined as an impaired ability to learn and remember new information (e.g. repetitive questions, misplacing personal items, forgetting events/appointments), and these deficits had to be considered to reflect a decline from a premorbid level of functioning. The diagnostic process primarily relied on medical history obtained from the patient and a reliable informant and neurological examination including cognitive screening. Additional diagnostic assessments, including imaging and/or neuropsychological assessments, were often conducted as part of clinical care or co-enrollment in research programmes. While these assessments supported clinical diagnoses, they did not determine it. We additionally excluded patients with insufficient pathology data, i.e. missing information about amyloid plaques, tau and Lewy bodies and LATE-NC. The primary analyses only considered patients with a pathological diagnosis of ADNC, LATE-NC or comorbid ADNC/LATE-NC. This resulted in a final sample of 165 patients from the Mayo cohort and 53 patients from the ADNI cohort. The workflow of patient inclusion is found in Supplementary Fig. 1. Analyses considering all primary neuropathological diagnoses associated with a progressive and predominant amnestic syndrome were conducted separately and are in Supplementary material (referenced in-text where appropriate). Data from 112 age- and sex-matched cognitively unimpaired (CU) controls from the Mayo Clinic Study of Aging were collected for imaging comparison purposes (MRI, FDG-PET) in the Mayo cohort. CUs had to be amyloid- and tau-negative based on PET imaging and have MRI and FDG-PET imaging available for inclusion in the study.

Patients and/or their legal representative provided written consent for their data to be used for research purposes. This study met HIPAA guidelines and was approved by the Mayo Clinic Institutional Review Board.

### Neuropathological assessment

Assessments for the Mayo and ADNI cohorts were performed by experienced neuropathologists in accordance with current diagnostic protocols^39^ and are described in Supplementary material. ADNC was diagnosed according to the ABC ranking score^40^ which includes the Thal staging of amyloid plaques, Braak staging of neurofibrillary tangles^41^ and the density measurement of neuritic plaques.^39^ A stage of Braak IV or less in the absence of significant amyloidosis (<2 Thal stage) was classified as primary age-related tauopathy (PART).^42^ In the Mayo cohort, TDP-43 type A was defined as TDP-43 immunoreactive neuronal cytoplasmic inclusions, dystrophic neurites and neuronal intranuclear inclusions in vulnerable cortical and subcortical areas. TDP-43 type B had predominantly neuronal cytoplasmic inclusions.^43^ Similar procedures were applied in ADNI (see https://adni.loni.usc.edu/methods/neuropath-methods/). TDP-43 staging was classified as FTLD-TDP-43–related or not (i.e. LATE-NC) based on its spatial distribution. LATE-NC staging was done in 40/90 of patients with confirmed TDP-43 from the Mayo cohort and all patients from the ADNI cohort. Lewy body disease (LBD) was staged according to published criteria,^44^ and significant burden was considered when pathology was documented in limbic and/or neocortical areas. Corticobasal degeneration was diagnosed by the presence of cortical and subcortical neuronal and glial lesions (i.e. astrocytic plaques) and thread-like processes in grey and white matter.^45^ Progressive supranuclear palsy staging was performed according to published criteria.^46^ Corticobasal degeneration and progressive supranuclear palsy were categorized as ‘FTLD-tau’. A diagnosis of argyrophilic grain disease was made if there were silver and tau-positive spindle-shaped lesions, coiled bodies and balloon neurons in trans-entorhinal and entorhinal cortex, amygdala or cingulate gyrus.^16^ The presence of other pathologies including hippocampal sclerosis and vascular disease (cerebral amyloid angiopathy, infarcts and lacunes, microbleeds, haemorrhages, arteriolosclerosis) was also assessed.

### Imaging acquisition and processing

Acquisition protocols for MRI and PET images are in Supplementary material. Regional MRI and FDG-PET data were generated for both the Mayo and ADNI cohorts using in-house processing pipelines from Mayo Clinic using Statistical Parameter Mapping 12 (SPM12). PET images were co-registered to their corresponding MRI image. All images were normalized into the Mayo Clinic Adult Lifespan Template and smoothed with a 6-mm full-width at half-maximum smoothing kernel. FDG-PET images were normalized to the pons to yield regional standard uptake value ratios (SUVRs). The FDG-PET IMT ratio was derived by dividing SUVR values from the inferior temporal lobe by the size-weighted sum of SUVR values from the amygdala and hippocampus,^31,47^ where higher values are indicative of LATE (thresholding procedures are described in the ‘Statistical analyses’ section).

Hippocampal volume calculation is described in separate publications.^48,49^ Briefly, it was corrected for intracranial volume by calculating the residuals from a linear regression based on a sex-specific formula. This is similar to the approach from Jack *et al*.,^49^ except for the use of SPM12 instead of FreeSurfer and a different CU sample as described in Stricker *et al*.^45^ This measure was combined across hemispheres.

Different methods were used to determine abnormality thresholds for global FDG-PET, amyloid-PET and tau-PET SUVR meta-regions of interest (meta-ROIs) across the Mayo and ADNI cohorts due to differences in processing pipelines, radiotracers for amyloid-PET and cohorts studied. This was done only for global measures, whereas regional data were generated using the same pipeline as described above. Briefly, global FDG-PET (normalized to the pons), amyloid-PET and tau-PET (normalized to the cerebellar crus) were derived from established meta-ROIs.^50-52^ Images from the Mayo cohort processed with the pipeline described above were used, and abnormality thresholds set at ≤1.47 (FDG-PET), ≥1.48 (amyloid-PET; PiB) and ≥1.29 (tau-PET; flortaucipir) according to published methods.^50,52^ Images from the ADNI cohort processed in Berkeley (CA, USA) using published methods^53^ were used. Abnormality thresholds were set at ≤1.21 (FDG-PET), ≥1.11 (amyloid-PET; florbetapir), ≥1.0818 (amyloid-PET; florbetaben) and ≥1.29 (tau-PET; flortaucipir) according to published methods.^51,53,54^ Amyloid-PET values from both cohorts were transformed into centiloids for descriptive purposes.

### Fluid biomarkers

CSF samples from both the Mayo and ADNI cohorts were analysed using published protocols.^55,56^ Briefly, samples were analysed using Elecsys β-Amyloid (1-42) CSF, Total-Tau CSF and Phospho-Tau (181P) CSF electrochemiluminescence immunoassays (Roche Diagnostics). Quality control procedures and technical limits were handled as previously described.^55^ Thresholds were set at ≤1026 pg/ml for Aβ42, ≥22 pg/ml for P-tau and ≥0.023 for the Aβ42/P-tau ratio.^55,57,58^

Procedures for the analysis and quality control of plasma assays for the Mayo cohort are described in a separate publication.^59^ Briefly, plasma phosphorylated-Tau 181 (pTau181) was measured with the Simoa® pTau-181 Advantage V2 kit following instructions from the manufacturer and ran on a Quanterix HD-X Analyzer (Quanterix, Lexington, MA, USA). The pTau181 threshold was set at <2.56 pg/ml according to previous research assessing the relationship between pTau181 and ADNC.^59^ Plasma assays in ADNI were collected and processed according to published methods^60,61^ and were measured using an in-house assay as previously described.^62^ Plasma pTau181 was measured with Simoa HD-X instruments (Quanterix, Billerica, MA, USA) as described elsewhere.^63^ The pTau181 threshold was set at <17.7 pg/ml according to previous research assessing the relationship between pTau181 and Alzheimer’s disease biomarkers.^63^ Of note, differences in pTau181 thresholds across the Mayo and ADNI cohorts are due to differences in scaling, threshold determination methods and assays (as noted above).

### Operationalization of the LANS criteria

The operationalization of the LANS criteria involves *in vivo* clinical, imaging and biomarker data. These operationalized criteria are not meant to be concretely applied to clinical practice; rather, their purpose is to provide construct validity to our proposed LANS criteria. In particular, the assessment of imaging criteria should be based on visual read in clinical practice as generally done in clinical neurology and radiology rather than quantitative methods as described below. These operationalized criteria are as follows:

1. Older age at evaluation (standard feature): age of ≥75 and older. Age at first visit was used. While we used 75 years old as threshold to operationalize this criterion in the context of this study, this is not a rigid cut-off for its application in clinical practice. Rather, older age increases the likelihood that the amnestic syndrome is caused by the degeneration of the limbic system.
2. Mild clinical syndrome (standard feature): diagnosis of mild cognitive impairment or mild amnestic dementia [i.e. score ≤4 on the Clinical Dementia Rating Sum of Boxes (CDR-SB)].^64^ The score at the first visit was used.
3. Hippocampal atrophy out of proportion to syndrome severity (standard feature): hippocampal volume smaller than expected according to the CDR-SB score (see procedure in ‘Statistical analyses’). The hippocampal volume at the last MRI was used.
4. Mildly impaired semantic memory (standard feature): given the less well-established literature in the context of LATE-NC, this feature is not operationalized in this iteration of the LANS criteria and is not included in the calculation of likelihoods in the context of the current study. However, its use is encouraged in clinical practice using expert clinical judgement.
5. Limbic hypometabolism (advanced feature): above-threshold value on the FDG-PET IMT ratio (see procedure in ‘Statistical analyses’). IMT ratio at the last FDG-PET was used.
6. Absence of neocortical degenerative disease pattern (advanced feature): above-threshold value on an established FDG-PET Alzheimer’s disease meta-ROI, which is indicative of an absence of the ADNC pattern. The score at the last FDG-PET was used. We used a meta-ROI in the context of this study, but this applies to any imaging biomarker indicative of neocortical degeneration, regardless of underlying aetiology.
7. Low likelihood of significant neocortical tau pathology (advanced feature): multiple scenarios are possible and are prioritized as follows: (i) negative amyloid-PET SUVR as it can serve as a surrogate marker of absence of neocortical tau^64^; (ii) positive amyloid-PET SUVR and negative tau-PET SUVR; (iii) negative CSF biomarkers for Alzheimer’s disease pathology if PET imaging is not available; and (iv) negative plasma biomarkers for Alzheimer’s disease pathology if PET imaging and CSF are not available.

To add context to the assessment of neocortical tau, the rationale of this criterion is to reduce the likelihood that the clinical syndrome is primarily driven by neocortical pathology as seen in the multi-domain forms of Alzheimer’s disease.^65,66^ The specification of ‘neocortical’ tau as opposed to tau in general is to account for the limbic variant of Alzheimer’s disease wherein associated clinical and pathologic findings localize to the limbic system,^67,68^ thus qualifying for LANS. Another point that deserves mention is that while we used a tau-PET SUVR that is highly associated with neocortical tau in this study,^50,53^ visual assessment as recommended by the FDA guidelines is recommended in clinical practice, which includes the posterior lateral temporal lobe as the only temporal region to count towards neocortical tau positivity (see https://www.accessdata.fda.gov/drugsatfda_docs/label/2020/212123s000lbl.pdf). In regard to plasma biomarkers, current assays are mostly sensitive to amyloid pathology, and current thresholds are not useful in determining limbic versus neocortical tau.^69^ The same applies to the CSF P-tau/Aβ ratio. Fluid biomarkers, in their current state of development, should only be used to rule out amyloidosis. If positive, these LANS criteria are not met, and further workup is recommended to investigate the presence of neocortical versus limbic distribution of tau.

### Statistical analyses

Statistical analyses were performed using R version 4.2.3. One-way ANOVAs and *χ*^2^ tests were used to assess between-group differences on demographic, clinical and biomarker data, and *post hoc* analyses were performed when the omnibus test was significant.

We compared FDG-PET and MRI findings between ADNC, ADNC/LATE-NC and LATE-NC patients and CUs from the Mayo cohort in a pairwise fashion using SPM12, resulting in t-maps. We applied a false discovery rate correction to control for multiple comparisons at the peak level. Cluster-level correction does not apply since we used unthresholded maps.

The procedure to assess disproportionate hippocampal atrophy according to clinical severity consisted of fitting a mixed linear model with CDR-SB as a predictor of hippocampal volume accounting for intra-individual change in the ADNI cohort. This was done including all patients with an amnestic syndrome with available CDR-SB and hippocampal volume, regardless of underlying pathology (*n* = 85, 435 time points). We then calculated the scaled residuals between predicted and true values. We used a receiver operating characteristic (ROC) curve analysis on *Z*-scored residuals to determine the optimal threshold for discriminating patients with LATE-NC (LATE and ADNC/LATE-NC) from those without LATE-NC (ADNC, ADNC/LBD, LBD, PART) based on the last MRI scan obtained. We applied this threshold in the ADNI cohort and in the Mayo cohort after predicting the residuals using the model defined in ADNI. We used a similar ROC curve analysis in the ADNI cohort to determine the FDG-PET IMT threshold that best discriminates individuals with LATE-NC (LATE and ADNC/LATE-NC) from those without LATE-NC (ADNC, ADNC/LBD, LBD, PART) based on the last FDG-PET scan obtained (*n* = 63). We then applied this threshold in the ADNI and Mayo cohorts.

Counts for standard and advanced LANS features and likelihoods derived from the operationalized criteria were measured for each patient with LATE-NC, ADNC/LATE-NC and ADNC. *χ*^2^ analyses followed by *post hoc* tests were conducted to assess the distribution of pathological diagnoses within likelihoods and the distribution of likelihoods within pathological diagnoses. This was done while combining highest and high likelihoods within a single category. We then fit a mixed linear model with an interaction between time from baseline (in years) and likelihood group (highest, high, moderate, low) as predictor and CDR-SB as outcome measure accounting for intra-individual change to assess differences in clinical progression across likelihoods. We then assessed FDG-PET differences between likelihood categories (highest/high, moderate, low) and CUs in pairwise fashion as described above.

We fit a logistic regression model in the Mayo cohort to perform a binary classification of patients with LATE-NC (i.e. LATE-NC and ADNC/LATE-NC) versus ADNC using LANS features as input. Only the tau score was considered as a categorical variable (positive, negative), whereas other variables (age at examination, CDR-SB score, hippocampal volume, IMT ratio, FDG-PET meta-ROI) were considered as continuous. We then performed out-of-sample predictions in the ADNI cohort using the model fitted in the Mayo cohort.

We performed an exploratory analysis specifically in patients with ADNC/LATE-NC from Mayo Clinic on the premise that some of these patients may have LATE-NC as a primary pathology and ADNC as a secondary pathology, and vice versa. We stratified ADNC/LATE-NC patients into highest/high, moderate and low LANS likelihoods. We fit a mixed linear model with an interaction between time from baseline (in years) and group (ADNC, ADNC/LATE-NC highest/high, ADNC/LATE-NC moderate, ADNC/LATE-NC low, LATE-NC) as predictor and CDR-SB as outcome measure accounting for intra-individual change to assess differences in clinical progression across groups. We then assessed FDG-PET differences between ADNC/LATE-NC likelihood groups and CUs in a pairwise fashion as described above.

## Results

### Sample characteristics

Demographic, clinical and biomarker data at baseline for patients with ADNC, LATE-NC and ADNC/LATE-NC from the Mayo and ADNI cohorts are displayed in Table 1. The data for all pathological diagnoses are displayed in Supplementary Table 1. LATE-NC and ADNC/LATE-NC patients were older than ADNC patients at presentation and death in the Mayo cohort only. In both cohorts, there were more *APOE4* carriers in ADNC and ADNC/LATE-NC groups compared to LATE-NC, and ADNC and ADNC/LATE-NC groups had higher amyloid-PET centiloid values compared to LATE-NC. ADNC/LATE-NC patients had, on average, higher baseline CDR-SB scores than LATE-NC patients in the ADNI cohort only. There were no other differences.

**Table 1:** Demographic, clinical and biomarker data of the Mayo and ADNI cohorts

Mayo cohort				
	ADNC	ADNC/LATE-NC	LATE-NC	*P*
*n*	75	81	9	
Age at first visit	74.2 (63.7, 80.5)	79 (72.2, 83.9)	86.2 (83.6, 93.3)	<0.001
Age at death	82.4 (71.1, 88.3)	89.1 (83.9, 92.4)	91.6 (88.7, 95.5)	<0.001
Sex (F, M)	33, 42	36, 45	4, 5	0.8
Education	16 (12,18)	16 (12, 17)	14 (13, 16)	0.38
CDR-SB	1.5 (0.5, 3.5)	1.5 (0.5, 3)	0.5 (0.5, 1.25)	0.39
AD dementia	27	30	1	
Amnestic MCI	48	51	8	
APOE4 (+, −)	47, 27	55, 24	1, 7	0.006
Amyloid-PET centiloid	110 (87.3, 129)	110 (76.7, 122)	9.75 (7.60, 13.4)	<0.001
AV1451 SUVR	1.85 (1.72, 2.28)	2.14 (1.40, 2.36)	1.26^a^	

ADNI cohort				
	ADNC	ADNC/LATE-NC	LATE-NC	*P*
*n*	26	19	8	
Age at first visit	75.8 (70.3, 81.1)	77.7 (72.2, 85.5)	74.9 (72.6, 81.7)	0.4
Age at death	79 (76.2, 85.8)	84 (79, 89)	80.5 (79.8, 87)	0.17
Sex (F, M)	9, 17	5, 14	1, 7	0.5
Education	16 (16, 18)	16 (13.5, 17)	16 (14.5, 18)	0.28
CDR-SB	2 (1, 3.38)	3.5 (2, 4.5)	1 (0.875, 1.12)	0.019
AD dementia	9	11	0	
Amnestic MCI	17	8	8	
APOE4 (+, −)	20, 6	13, 6	1, 7	0.004
Amyloid-PET centiloid	69.5 (50.2, 110)	91 (81, 102)	−5 (−11.8, 0)	0.0015
AV1451 SUVR	NA	1.52 (1.49, 1.55)	1.08^a^	

CDR-SB, Clinical Dementia Rating Scale Sum of Boxes; ADNCs, Alzheimer’s disease neuropathological changes; LATE-NCs, limbic-predominant age-related TDP-43 encephalopathy neuropathological changes; MCI, mild cognitive impairment; SUVR, standard uptake value ratio.

^a^One observation only. Values are expressed as counts or median and interquartile ranges.

**Figure 1:**
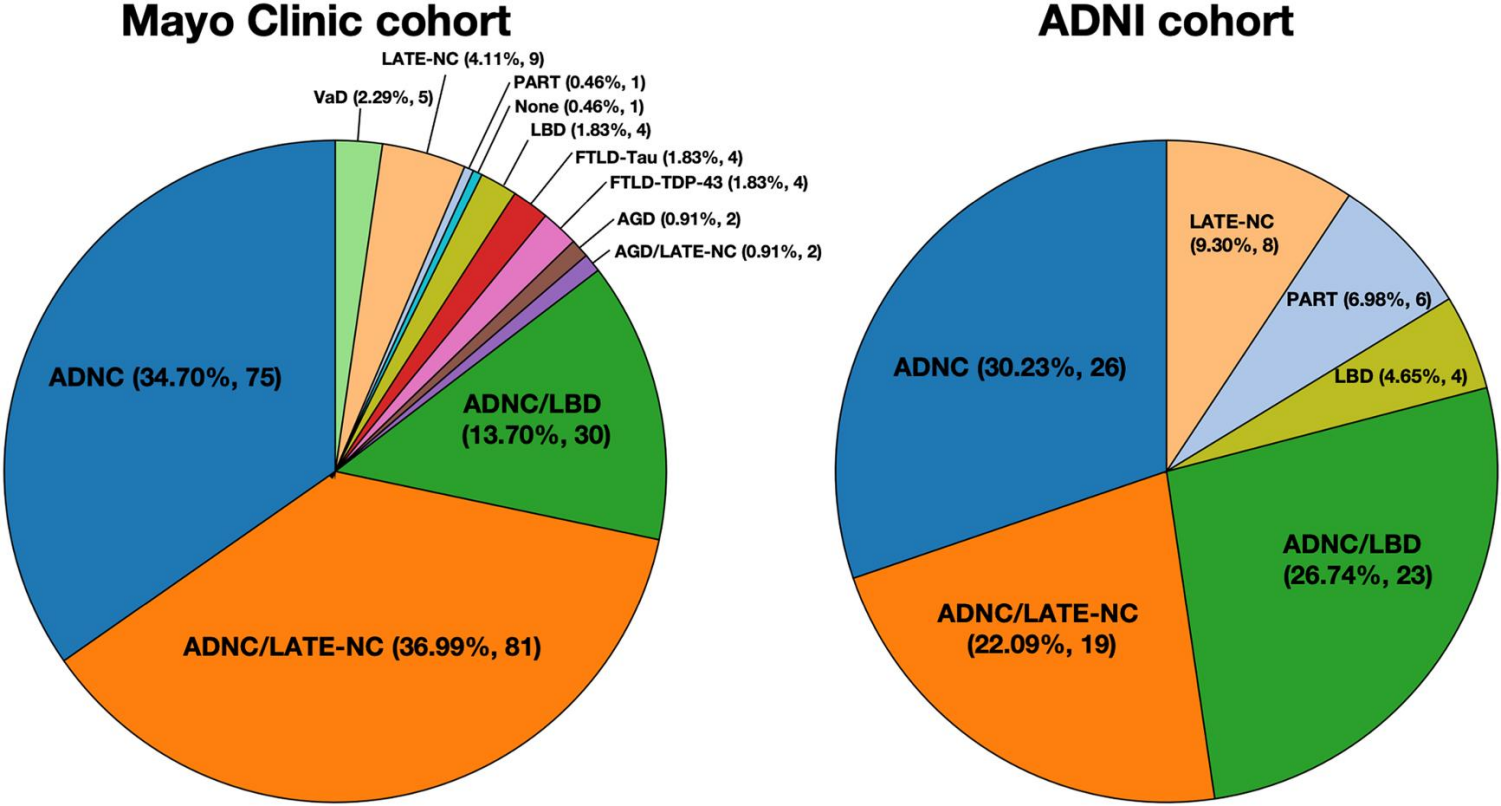
**Primary neuropathological diagnoses associated with a predominant and progressive neurodegenerative amnestic syndrome in the Mayo and ADNI cohorts.** ADNCs, Alzheimer’s disease neuropathological changes; LATE-NCs, limbic-predominant age-associated TDP-43 encephalopathy neuropathological changes; LBD, Lewy body disease; AGD, argyrophilic grain disease; FTLD, fronto-temporal lobar degeneration; PART, primary age-related tauopathy; VaD, vascular disease.

The distribution of pathological diagnoses underlying a progressive and predominant neurodegenerative amnestic syndrome in the Mayo and ADNI cohorts is displayed in Fig. 1. ADNC, ADNC/LATE-NC and LATE-NC accounted for 35, 37 and 4% of cases in the Mayo cohort, respectively, and 30, 22 and 9% of cases in the ADNI cohort, respectively. Vascular pathologies (cerebral amyloid angiopathy, infarcts/lacunes, microbleeds, haemorrhages, arteriolosclerosis) were not considered in primary pathological diagnoses unless they were the only significant pathological feature (i.e. vascular dementia). The presence and severity of vascular pathologies across pathological diagnoses are listed in Supplementary Table 2.

### FDG-PET and MRI findings

**Figure 2:**
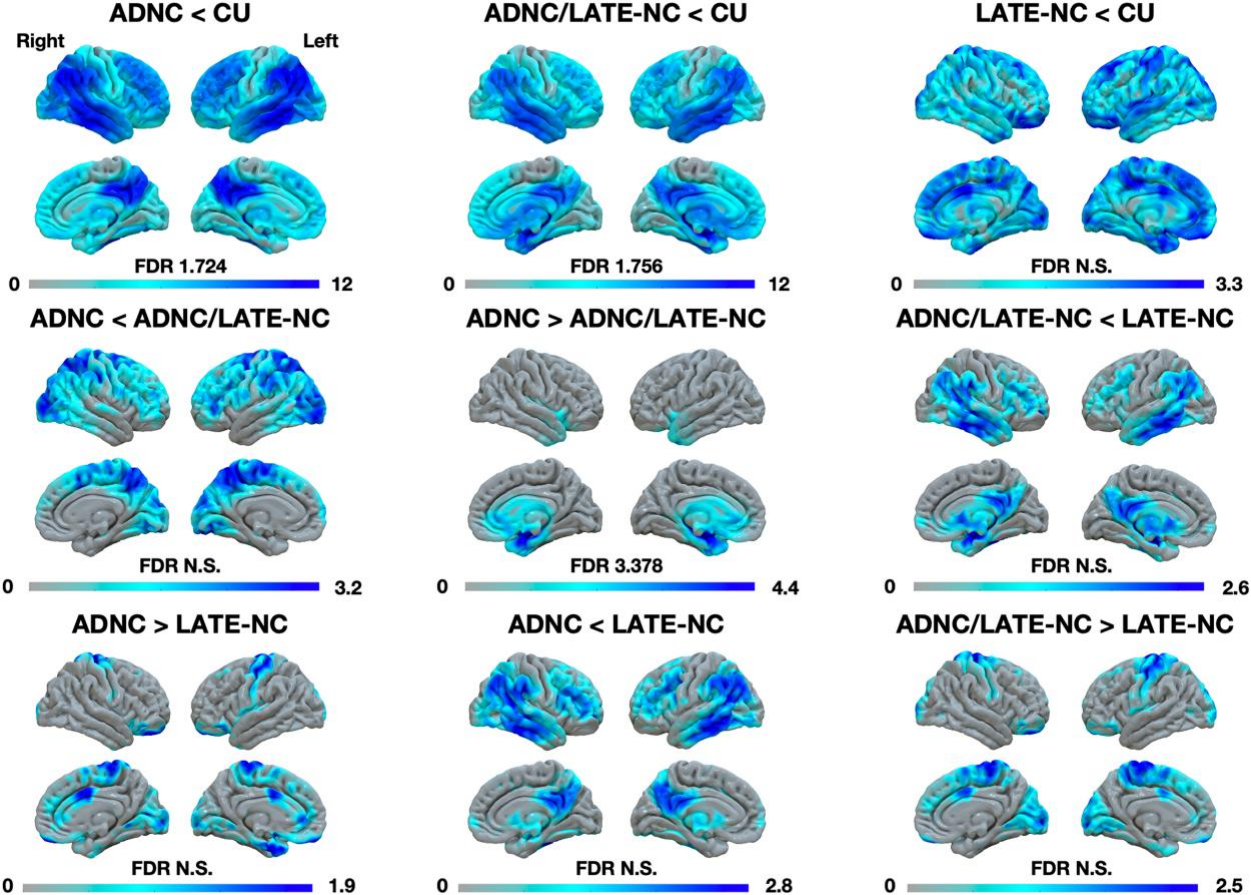
**FDG-PET findings between ADNC, ADNC/LATE-NC, LATE-NC and CU controls.** The ‘less than’ sign reflects less metabolism in a given group relative to the other and vice versa. Contrasts were generated using SPM12 using pairwise *t*-comparisons between groups at the voxel level and involved 53 ADNC cases, 33 ADNC/LATE-NC cases, 4 LATE-NC cases and 112 CUs. ADNCs, Alzheimer’s disease neuropathological changes; LATE-NCs, limbic-predominant age-associated TDP-43 encephalopathy neuropathological changes; CU, cognitively unimpaired; FDR, false discovery rate; N.S., non-significant; FDG-PET, fluorodeoxyglucose-PET.

FDG-PET and MRI contrasts comparing patients groups to CUs showed patterns of degeneration in lateral temporal and hippocampal areas in ADNC/LATE-NC patients and in lateral temporo-parietal and precuneus areas in ADNC. FDG-PET findings are displayed in Fig. 2, and MRI findings are displayed in Supplementary Fig. 2. Only one contrast between patient groups survived correction for multiple comparisons for both MRI and FDG-PET modalities. This contrast revealed that ADNC/LATE-NC had significantly more temporo-limbic degeneration involving the hippocampal, insular, temporo-polar, middle frontal and orbitofrontal areas compared to ADNC patients. Comparisons involving LATE-NC patients failed to reveal significant differences at the group level, likely owing to the small sample size.

### LANS likelihood differences

Table 2 indicates the distribution of ADNC, LATE-NC and ADNC/LATE-NC patients across stand-alone LANS features and LANS likelihoods for the Mayo and ADNI cohorts. These data for all pathological diagnoses are displayed in Supplementary Table 3. The assessment of differences in the distribution of LANS likelihood only applied to patients for whom all LANS features were available. Given the small numbers in each group, especially LATE-NC, we emphasize the qualitative assessment of frequencies of pathological diagnoses across LANS likelihoods, although inferential statistics have also been performed.

**Table 2:** LANS features and likelihoods across pathological diagnoses for the Mayo and ADNI cohorts

Standard and advanced LANS features (meets feature/total)						
Mayo cohort	Age ≥75	Mild syndrome	Hippocampal atrophy	Limbic hypometabolism	Absence of neocortical hypometabolism	Low neocortical tau likelihood
ADNC	36/75	58/75	25/63	9/53	5/53	7/60
ADNC/LATE-NC	53/81	66/81	38/54	15/33	7/33	1/49
LATE-NC	8/9	8/9	3/4	3/4	1/4	5/7

LANS likelihoods (only including patients with all features available)				
Mayo cohort Diagnosis	Low	Moderate	High	Highest
ADNC	31	17	1	0
ADNC/LATE-NC	12	8	13	0
LATE-NC	0	0	2	2

Standard and advanced LANS features						
ADNI cohort	Age ≥75	Mild syndrome	Hippocampal atrophy	Limbic hypometabolism	Absence of neocortical hypometabolism	Low neocortical tau likelihood
ADNC	15/26	20/26	11/26	1/18	2/18	1/22
ADNC/LATE-NC	12/19	12/19	15/19	4/13	1/13	0/17
LATE-NC	4/8	8/8	5/8	5/6	1/6	8/8

LANS likelihoods (only including patients with all features available)				
Diagnosis	Low	Moderate	High	Highest
ADNC	11	7	0	0
ADNC/LATE-NC	8	3	1	0
LATE-NC	2	0	1	3

LANS, limbic-predominant amnestic neurodegenerative syndrome; ADNCs, Alzheimer’s disease neuropathological changes; LATE-NCs, limbic-predominant age-related TDP-43 encephalopathy neuropathological changes.

In the Mayo cohort, all LATE-NC (4/4) patients had a highest/high likelihood. Thirty-one of forty-nine (63%), 17/49 (35%) and 1/49 (2%) of ADNC patients had low, moderate and highest/high likelihoods, respectively. Twelve of thirty-three (36%), 8/33 (24%) and 13/33 (39%) of ADNC/LATE-NC patients had low, moderate and highest/high likelihoods, respectively. Assessments of the distribution of pathological diagnoses within likelihood categories revealed that there were proportionally more ADNC/LATE-NC than ADNC patients within the highest/high likelihood category (*P* = 0.007). There were more ADNC and ADNC/LATE-NC patients than LATE-NC patients within the moderate likelihood category (*P* < 0.001 and *P* = 0.02, respectively). There were more ADNC and ADNC/LATE-NC patients than LATE-NC patients (*P* = 0.004) and more ADNC patients than ADNC/LATE-NC patients (*P* < 0.001) within the low likelihood category. Assessment of the distribution of likelihood categories within pathological diagnoses revealed that there were more ADNC patients with low and moderate likelihoods compared to highest/high likelihoods (*P* < 0.001 and *P* = 0.002, respectively). There were no other differences.

In the ADNI cohort, 4/6 (67%) and 2/6 (33%) of LATE-NC cases had highest/high and low likelihoods, respectively. Eleven of eighteen (61%) and 7/18 (39%) of ADNC cases had low and moderate likelihoods, respectively. Eight of twelve (67%), 3/12 (25%) and 1/12 (8%) of ADNC/LATE-NC patients had low, moderate and highest/high likelihoods, respectively. Assessments of the distribution of pathological diagnoses within likelihood categories only revealed a trend towards more ADNC patients than LATE-NC patients within the moderate likelihood category (*P* = 0.09). Assessment of the distribution of likelihood categories within pathological diagnoses revealed that there were more ADNC patients with a low likelihood compared to highest/high likelihoods (*P* = 0.04). There were no other differences.

**Figure 3:**
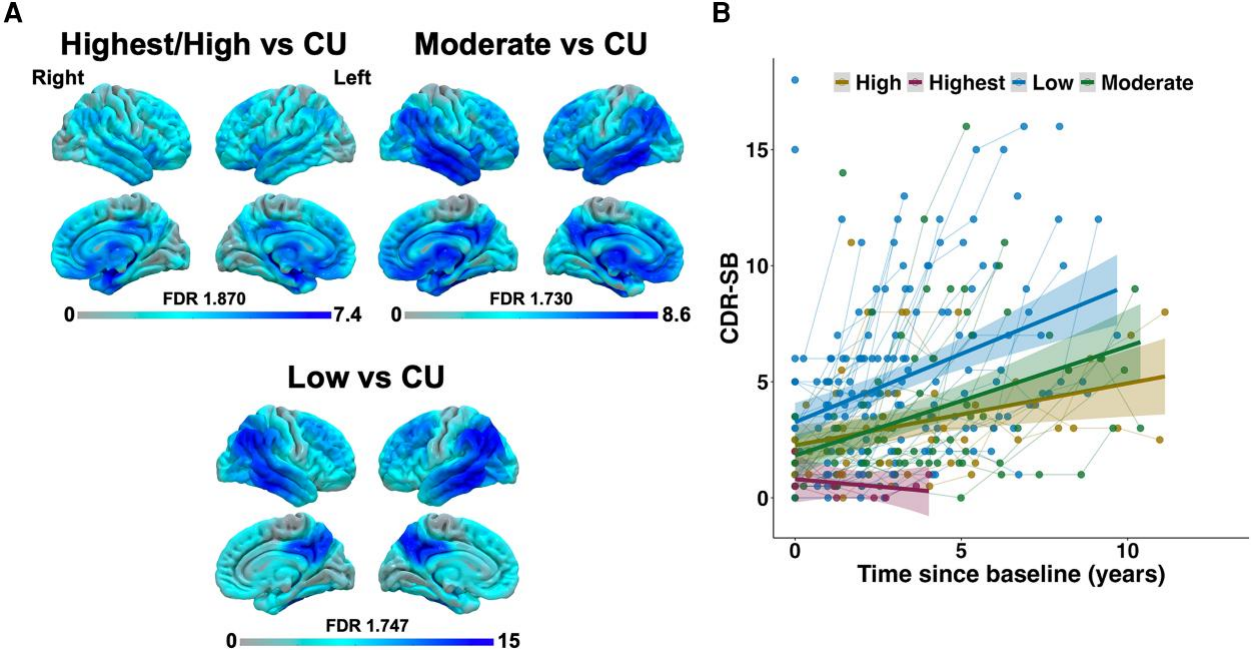
**FDG-PET and longitudinal CDR-SB findings across LANS likelihoods.** (**A**) FDG-PET findings comparing LANS likelihoods to CUs. Contrasts were generated using SPM12 using pairwise *t*-comparisons between groups at the voxel level and involved 42 cases with a low likelihood, 25 cases with a moderate likelihood, 15 cases with a highest/high likelihood and 112 CUs. (**B**) Longitudinal CDR-SB trajectories at the individual and group level are displayed on the right. CU, cognitively unimpaired; FDR, false discovery rate; CDR-SB, Clinical Dementia Rating Scale Sum of Boxes.

Assessment of longitudinal trajectories of CDR-SB according to LANS likelihood and FDG-PET findings comparing LANS likelihoods to CUs are displayed in Fig. 3. Results from mixed linear modelling between longitudinal CDR-SB and LANS likelihoods are in Supplementary Table 4. This analysis showed that all groups had equivalent CDR-SB scores at baseline. LANS likelihoods differed in their CDR-SB trajectory over time in that patients with a high likelihood had a slower increase of CDR-SB score than those with a low likelihood. There were trends towards significance for a slower increase of CDR-SB scores in patients with a high likelihood compared to those with moderate and low likelihoods.

FDG-PET findings showed that patients with highest/high likelihoods had patterns of degeneration mostly involving the temporo-limbic system and inferior frontal areas with little involvement of neocortical areas compared to CUs. In contrast, those with a low likelihood had more pronounced neocortical degeneration mostly involving lateral temporo-parietal areas with little involvement of the medial temporal lobe. The moderate likelihood showed a mixture of these patterns, with involvement of both medial temporo-limbic and neocortical areas.

### Binary classification of LATE-NC

A logistic model classifying patients with (LATE-NC, ADNC/LATE-NC) or without LATE-NC (ADNC) based on operationalized LANS criteria (age at examination, CDR-SB score, hippocampal volume, IMT ratio, FDG-PET meta-ROI, tau positivity) achieved a balanced accuracy of 74.6% in the Mayo cohort. The sensitivity and specificity of the model were 78.6 and 70.6%, respectively. There were 12/15 (80%) true positives (i.e. correctly classified with LATE-NC) and 11/16 (68.75%) true negatives (i.e. correctly classified without LATE-NC). Out-of-sample predictions in the ADNI cohort using the model fitted in the Mayo cohort achieved a balanced accuracy of 73.3% in the ADNI cohort. The sensitivity and specificity of the model were 60.9 and 85.7%, respectively. There were 9/15 (60%) true positives (i.e. correctly classified with LATE-NC) and 14/15 (93.33%) true negatives (i.e. correctly classified without LATE-NC). A qualitative assessment of age and CDR-SB scores in misclassified cases showed that false positive (predicted to have LATE-NC whereas they did not) were older and less impaired than ADNC cases (i.e. true negatives) and that false negatives (predicted not to have LATE-NC whereas they did) were younger and more impaired than ADNC/LATE-NC and LATE-NC cases (i.e. true positives). While these comparisons were not statistically significant, this suggests that these cases may have mixed clinical features and are not representative of the archetypical profiles of ADNC or LATE-NC, respectively.

### ADNC/LATE-NC heterogeneity

**Figure 4:**
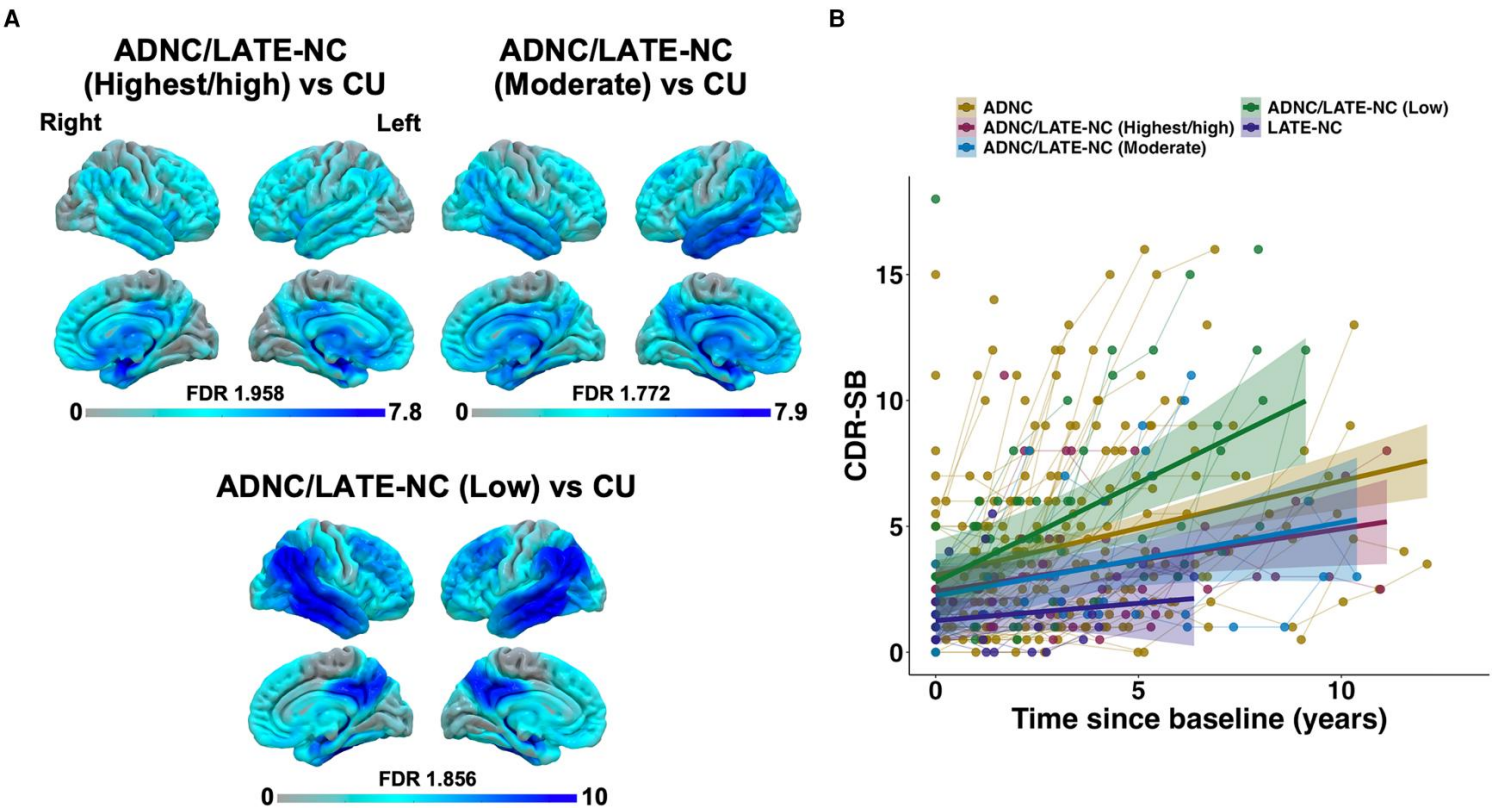
**FDG-PET and longitudinal CDR-SB findings in ADNC/LATE-NC according to LANS likelihoods relative to other groups.** (**A**) FDG-PET findings comparing ADNC/LATE-NC to CUs. Contrasts were generated using SPM12 using pairwise *t*-comparisons between groups at the voxel level and involved 12 ADNC/LATE-NC cases with a low likelihood, 8 ADNC/LATE-NC cases with a moderate likelihood, 13 ADNC/LATE-NC cases with a high/highest likelihood and 112 CUs. (**B**) Longitudinal CDR-SB trajectories at the individual and group level are displayed on the right. ADNCs, Alzheimer’s disease neuropathological changes; LATE-NCs, limbic-predominant age-associated TDP-43 encephalopathy pathological changes; CU, cognitively unimpaired; FDR, false discovery rate; CDR-SB, Clinical Dementia Rating Scale Sum of Boxes.

We conducted an exploratory analysis on the clinical heterogeneity of ADNC/LATE-NC patients on the premise that some have LATE-NC as a primary pathology and ADNC as a co-pathology and vice versa. We stratified ADNC/LATE-NC patients based on their LANS likelihood into highest/high (*n* = 13), moderate (*n* = 8) and low (*n* = 12) likelihoods. This was only done in the Mayo cohort given the relatively low heterogeneity of LANS likelihoods in ADNC/LATE-NC patients from the ADNI cohort. Results from these analyses are visually depicted in Fig. 4.

Results from the mixed linear model assessing the longitudinal trajectory of CDR-SB scores according to time since baseline across groups (ADNC, ADNC/LATE-NC highest/high, ADNC/LATE-NC moderate, ADNC/LATE-NC low, LATE-NC) are in Supplementary Table 5. This analysis showed that all groups had statistically equivalent CDR-SB scores at baseline. Groups differed in their CDR-SB trajectory over time in that ADNC/LATE-NC patients with a low likelihood had a steeper increase in CDR-SB scores compared to all other groups.

FDG-PET findings showed that ADNC/LATE-NC patients with highest/high likelihoods had patterns of degeneration mostly involving the temporo-limbic system with little involvement of neocortical areas aside from the inferior frontal areas compared to CUs. In contrast, ADNC/LATE-NC patients with a low likelihood had more pronounced neocortical degeneration mostly involving lateral temporo-parietal areas with lesser involvement of the medial temporal lobe. ADNC/LATE-NC patients with a moderate likelihood showed an intermediate pattern, with involvement of both medial temporo-limbic and neocortical areas.

## Discussion

We developed a set of clinical criteria to identify individuals with a predominant and progressive amnestic syndrome driven by degeneration of the temporo-limbic system. We termed this clinical entity LANS to emphasize that it does not necessarily have a strict mapping to LATE-NC even though they share limbic predominance. This neurologic condition is characterized by progressive limbic degeneration and associated clinical signs and symptoms requiring comprehensive evaluation including history, physical, neuropsychological assessment, imaging and fluid biomarkers. Various combinations of neuropathologies like LATE-NC, ADNC, LBD, argyrophilic grain disease and PART have been observed in the setting of this neurologic syndrome. We operationalized and validated the LANS criteria by tying limbic degeneration to an older age at evaluation, mild clinical syndrome, disproportionate hippocampal atrophy according to clinical severity, limbic hypometabolism, absence of neocortical degenerative disease pattern and low likelihood of neocortical tau. The LANS criteria effectively categorized LATE-NC, ADNC/LATE-NC and ADNC patients from two clinicopathological cohorts (Mayo and ADNI). Most patients with highest or high LANS likelihoods had post-mortem evidence of LATE-NC, most patients with moderate or low LANS likelihoods had evidence of ADNC, and those with evidence of ADNC/LATE-NC patients had a wider distribution of LANS likelihoods. Assessing clinical and imaging features across LANS likelihood regardless of underlying pathology showed that patients with higher likelihoods had a slower clinical course and patterns of temporo-limbic degeneration with minimal involvement of neocortical areas, while those with lower likelihoods showed the opposite pattern. Stratifying ADNC/LATE-NC patients according to their LANS likelihoods revealed a high degree of heterogeneity, where those with higher likelihoods had a slower clinical course and those with lower likelihood had the worse clinical prognosis compared to all other groups. The implementation of LANS criteria has critical implications for clinical and therapeutic endeavours as well as for a deeper understanding of the aetiologic landscape of predominant and progressive amnestic syndromes.

Patients with LATE-NC had a mild level of cognitive impairment at presentation with relatively slow progression of impairment over time, which further consolidates findings from separate studies findings a memory-dominant profile characterized by an indolent clinical course in LATE-NC.^1,3,6,70^ Interestingly, patients with ADNC/LATE-NC with a high LANS likelihoods showed a relatively similar profile in terms of clinical progression, which aligns with our hypothesis that these patients have LATE-NC as the primary driver of their clinical symptoms with ADNC as a secondary pathology. This is further supported by the finding of a more rapid clinical decline in ADNC/LATE-NC patients with a low LANS likelihood, which are hypothesized to have ADNC as the primary driver of their clinical symptoms and may thus have combined limbic and neocortical functional neurodegeneration. These findings bring nuance to the prevailing view that patients with evidence for both pathologies systematically have worse clinical outcomes than ADNC alone.^6,70^ They also allow for the prediction of the primary symptom-driving pathology *in vivo* in the context of comorbidity.

FDG-PET and MRI imaging results are also indicative of a selective degeneration of the limbic system including strong involvement of the hippocampus associated with LATE-NC, above and beyond the contribution of ADNC. In fact, ADNC was significantly associated with degeneration of the lateral temporal lobe and posterior neocortical areas rather than the limbic system. This aligns well with previous studies assessing imaging features of LATE-NC and/or ADNC.^24,25,28-31^

**Figure 5:**
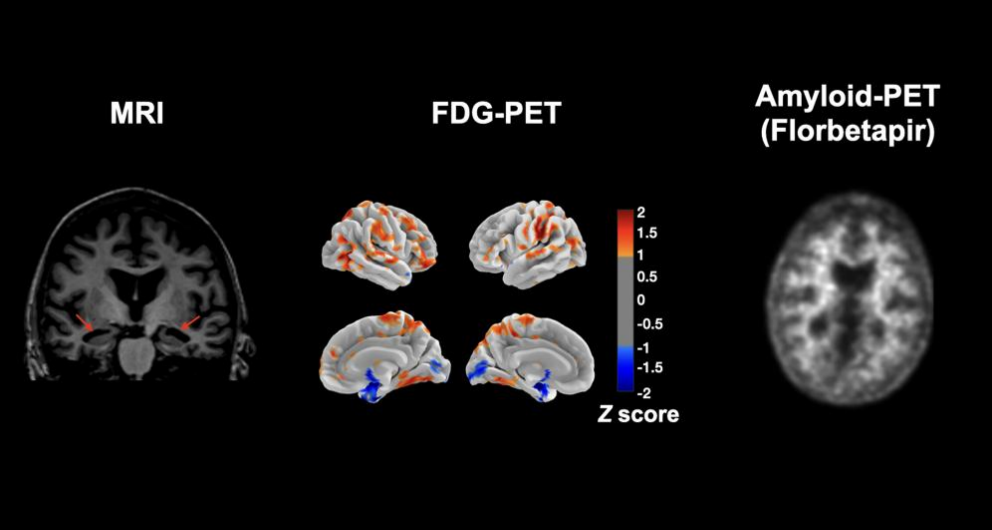
**Case example of a prospective use of the LANS criteria in clinical settings.** This patient was seen at Mayo Clinic, Rochester, to determine eligibility for an anti-amyloid monoclonal antibody therapy, and the LANS criteria were internally defined at the time of evaluation. The criteria were operationalized using clinical judgement of available data including visual reads of neuroimaging as is the current standard in clinical practice. This patient was a female over the age of 75 with a history of memory problems for several years. She lived alone, managed her instrumental activities of daily living independently and was still working part-time. Neuropsychological testing revealed moderate to severe impairment on measures of delayed recall and fragility of salient semantic knowledge (i.e. she could not recall details about the events occurring on 11 September 2001; she named another building than the World Trade Center and did not know how many buildings were hit). Performance was also mildly low on a task of object naming. The remainder of the assessment (i.e. global cognition, executive functions, visuospatial processing and language) was within or above expectations from a normative standpoint. The profile was consistent with single-domain amnestic mild cognitive impairment. MRI revealed disproportionate bilateral hippocampal atrophy, and she was assigned a moderate likelihood LANS diagnosis. Subsequently, a brain FDG-PET revealed prominent temporo-limbic and milder inferior frontal hypometabolism in the absence of a neocortical degenerative pattern by visual read. Amyloid-PET was read as negative. She thus met all core, standard and advanced criteria for LANS, corresponding to the highest likelihood. She did not meet criteria for an anti-amyloid monoclonal antibody therapy and was counselled about diagnosis, prognosis and most likely underlying aetiology.

The proposed LANS criteria have several clinical implications. The primary implication of these criteria is to accurately diagnose patients for whom the cause of progressive and predominant amnestic symptoms is linked to the degeneration of the limbic system with a high likelihood of LATE-NC as a symptom-driving pathology. It is noteworthy that the diagnosis of LANS is based on likelihoods, and not on definitive categories as seen with other classifications. This means that the unavailability of some features does not preclude rendering LANS diagnosis. Rather, it prevents from reaching higher LANS likelihoods and prompts further workup to ascertain that symptoms are caused by predominant limbic degeneration. This underlines the critical role of imaging and biomarkers to increase the diagnostic confidence of LANS. Figure 5 demonstrates a prospective example of clinical case of LANS, where a moderate likelihood was assigned based upon the assessment of core and standard features. The assessment of advanced features (negative amyloid-PET, predominant limbic hypometabolism on FDG-PET) increased confidence that limbic degeneration was the culprit of symptoms, thus allowing for the highest LANS likelihood.

The accurate diagnosis of LANS has the potential to improve prognostication and counselling of patients about the nature of their symptoms. Current evidence indicates that the symptomatology primarily involves episodic memory for recent events, while neocortical functions (e.g. executive functioning and visuospatial reasoning) are expected to remain relatively preserved throughout the disease course.^1,2,19^ An accurate diagnosis is also relevant for prognostication, as LANS has a likelihood of being associated with LATE-NC, which is associated with a relatively slow and milder clinical course compared to canonical Alzheimer’s disease–type dementia per our results and other studies.^1,3,6,70^ This is again exemplified in Fig. 5, where a highest LANS likelihood allowed to have precise diagnosis, counsel prognosis, guide treatment options and provide answers about the mostly likely underlying aetiology in this patient allowing for engagement with research and other health information on this topic. Without a LANS construct, we are left only saying that a patient does not qualify for anti-amyloid treatment and that they do not have Alzheimer’s disease. Having a positive diagnosis serves additional utility beyond that and facilitates comprehensive medical care.

To put this work in contexts, recent work on terminology would characterize LANS as a subtype of amnestic MCI or amnestic dementia.^34,35^ The addition of preservation of neocortical functions, older age at symptom onset, mild clinical severity and semantic memory impairment would make these amnestic syndromes more likely to be LANS and the additional imaging would localize the syndromes to the limbic system. These refinements would add greater specificity to the diagnosis for clinicians. LANS in the context of normal amyloid levels can also be examined through the lens of suspected non–Alzheimer disease pathophysiology, which refers to amyloid-negative, neurodegeneration-positive individuals.^71^ However, suspected non–Alzheimer disease pathophysiology encompasses a broader spectrum of conditions than LANS, as it is a biomarker-based definition regardless of clinical status.

The LANS criteria have critical therapeutic implications. This is especially true in this era of emerging anti-amyloid monoclonal antibody therapies.^32,33,72,73^ As these therapies make their way into clinical practice, the LANS criteria can be of relevance for identifying patients with a high likelihood of LATE-NC as the primary cause of their symptoms and guide clinical decision-making and counselling regarding potential therapeutic avenues. It is important to mention that positive Alzheimer’s disease biomarkers do not rule out a diagnosis of LANS, as a progressive and predominant amnestic syndrome caused by LATE-NC or other pathologies can be observed in concomitance with incidental amyloidosis or ADNC.^30,31^ The use of the LANS likelihoods can help determine whether the amnestic syndrome is most likely driven by a primary non–Alzheimer disease pathophysiology with secondary ADNC or vice versa, as demonstrated by our analysis deciphering the heterogeneity of patients with ADNC/LATE-NC. It is important to mention that we do not make treatment recommendations based on this work, but the LANS criteria allow to study such a question. Future studies will be important to determine how patients with positive Alzheimer’s disease biomarkers respond to disease-modifying therapies according to their LANS likelihood. Finally, the LANS criteria are a major advancement in the overarching goal of tying an *in vivo* neurologic syndrome to underlying LATE-NC. The other critical component needed to achieve this is the development of *in vivo* diagnostic biomarkers of TDP-43 specific for LATE-NC. PET, CSF and biofluid biomarkers of TDP-43 are hopefully on the horizon.^1,74,75^ This would allow for defining the clinical entity of LATE as a high-likelihood LANS syndrome with biomarker evidence of LATE-NC. Defining such a clinicopathological entity will be important for the design of clinical trials aimed at LATE-NC in terms of defining eligibility criteria and outcomes measures.

It is important to reiterate that while LANS is highly associated with LATE-NC, it can be associated with other pathologic entities that selectively target the limbic system. Supplemental analyses provide some evidence that pathologies associated with a predominant amnestic syndrome other than LATE-NC may, in rare instances, have a high LANS likelihood (e.g. LBD, argyrophilic grain disease, advanced PART). One example that can be a potential source of clinical conundrums is the limbic variant Alzheimer’s disease, where tau predominantly localizes to the limbic system and therefore qualifies for LANS. In this scenario, the advanced LANS criteria in combination with visual assessment of tau-PET can help in determining which pathology has the highest likelihood of driving clinical symptoms.

Notably, there were slightly lower LANS likelihoods in ADNC/LATE-NC patients in the ADNI cohort relative to the Mayo cohort. This is likely due to discrepancy in recruitment and sampling strategies. The Mayo cohort draws from clinical practice in a tertiary behavioural neurology clinic (Alzheimer’s Disease Research Center) and randomly selected individuals living in Olmsted County, MN, USA (Mayo Clinic Study of Aging).^76^ It is thus designed to reflect a combination of the clinicopathologic variability encountered in Alzheimer’s disease–oriented clinical context and with more representative settings. On the other hand, ADNI was designed as a research cohort aimed at the clinical and biological characterization of individuals on the clinicopathologic spectrum of Alzheimer’s disease.^77,78^ It is not meant to reflect the heterogeneity encountered in clinical practice. These fundamental differences may explain the relatively younger age of LATE-NC patients and the lower frequency of ADNC/LATE-NC with a high LANS likelihood. We believe the performance of the LANS criteria in the Mayo cohort is a more accurate reflection of how the LANS criteria are expected to perform in clinical settings. It is also important to mention that we undertook several steps to avoid overfitting the LANS criteria validation in the Mayo cohort. This includes the use of externally validated thresholds for most imaging and tau biomarkers (e.g. CSF, ptau181, molecular PET imaging) and deriving optimal thresholds for hippocampal atrophy and the IMT ratio in the ADNI cohort rather than Mayo.

There are some limitations to this work. An evident caveat is the low number of patients with LATE-NC without ADNC. The lack of statistical differences between these patients and other groups in terms of clinical and imaging features could be attributable to the small sample size rather than a genuine absence of differences. However, most patients with LATE-NC had highest and high LANS likelihoods, suggesting that our proposed criteria effectively identify these individuals. Although the proposed LANS criteria include impaired semantic memory, we did not incorporate this feature in the operationalized criteria given the less robust and variably defined evidence of such impairment. Efforts are underway to better characterize the nature and extent of semantic memory impairment in patients with LATE-NC and develop cognitive tests sensitive to such impairment. This should not, however, prevent clinicians from considering this feature in their clinical decision-making when LANS is in the differential, as highlighted in the case example in Fig. 5. The classification model based on the features of the LANS criteria had modest accuracy in distinguishing LATE-NC from ADNC at autopsy, which may be due to a range of factors including increased pathological burden in late-stage disease and partially overlapping clinical and radiological features between the two pathological entities. Prospective studies are required to further assess the value of the LANS criteria in predicting underlying pathologies predominantly driving limbic versus neocortical degeneration. This study is retrospective in nature. The implementation of the LANS criteria in clinical settings and prospective studies are needed to further validate and refine this set of criteria. Finally, while we report vascular burden across both cohorts, this information was not utilized in assigning pathological diagnoses unless seen in isolation. Future work with sample allowing for such stratification is required to assess how vascular damage influences the clinical trajectory and pathophysiology of LANS.

In conclusion, we developed and validated a set of criteria for LANS designed to be used in clinical practice to identify individuals with a predominant amnestic syndrome driven by the degeneration of the temporo-limbic system with a high likelihood of underlying LATE-NC. This has important clinical implications including differential diagnosis with other common causes of episodic memory impairment such as neocortical degeneration driven by ADNC, counselling patients about the nature and course of their symptoms, providing appropriate treatments, referral to research programmes and the development of therapeutic efforts aimed at LATE-NC. Several steps lay ahead to improve the definition of LANS including the conduction of prospective studies and the development of clinical tools that are sensitive and specific to its cognitive features. Finally, the development of *in vivo* diagnostic markers of TDP-43 pathology is needed to embed LANS into a clinicopathological entity driven by LATE-NC, i.e. LATE.

## Supplementary Material

fcae183_Supplementary_Data

## Data Availability

Data from the Mayo Clinic Study of Aging and the Mayo Clinic Alzheimer’s Disease Research Center are available upon request (https://www.mayo.edu/research/centers-programs/alzheimers-disease-research-center/data-requests). ADNI data can be downloaded at https://adni.loni.usc.edu/. The Mayo Clinic Adult Lifespan Template can be found at https://www.nitrc.org/projects/mcalt/. The R code for the statistical analyses can be found on https://github.com/nickcorriveaul/R_scripts_manuscripts.
